# Pro-arrhythmogenic effects of *CACNA1C* G1911R mutation in human ventricular tachycardia: insights from cardiac multi-scale models

**DOI:** 10.1038/srep31262

**Published:** 2016-08-09

**Authors:** Jieyun Bai, Kuanquan Wang, Qince Li, Yongfeng Yuan, Henggui Zhang

**Affiliations:** 1 School of Computer Science and Technology, Harbin Institute Technology, Harbin, 150001, China; 2 Biological Physics Group, School of Physics and Astronomy, University of Manchester, Manchester, M13 9PL, UK

## Abstract

Mutations in the *CACNA1C* gene are associated with ventricular tachycardia (VT). Although the *CACNA1C* mutations were well identified in patients with cardiac arrhythmias, mechanisms by which cardiac arrhythmias are generated in such genetic mutation conditions remain unclear. In this study, we identified a novel mechanism of VT resulted from enhanced repolarization dispersion which is a key factor for arrhythmias in the *CACNA1C* G1911R mutation using multi-scale computational models of the human ventricle. The increased calcium influx in the mutation prolonged action potential duration (APD), produced steepened action potential duration restitution (APDR) curves as well as augmented membrane potential differences among different cell types during repolarization, increasing transmural dispersion of repolarization (DOR) and the spatial and temporal heterogeneity of cardiac electrical activities. Consequentially, the vulnerability to unidirectional conduction block in response to a premature stimulus increased at tissue level in the G1911R mutation. The increased functional repolarization dispersion anchored reentrant excitation waves in tissue and organ models, facilitating the initiation and maintenance of VT due to less meandering rotor tip. Thus, the increased repolarization dispersion caused by the G1911R mutation is a primary factor that may primarily contribute to the genesis of cardiac arrhythmias in Timothy Syndrome.

Ventricular tachycardia (VT) characterized by high rates of ventricular excitation may cause sudden cardiac death^1^. Thus, it is important to understand the mechanisms of VT for practical cardiology. Although structural cardiac disease and electrical remodeling of ion channels were documented to be major contributory factors of initiating and sustaining VT^2^,^3^,^4^, the underlying mechanisms of VT are as yet incompletely understood. However, VT patients in some instances have characteristic solitary gene defects without other structural diseases^3^. In particular, the long-QT syndrome (LQTS), associated with genetic disorders, is characterized by ion channel remodeling, which may produce heterogeneous action potential (AP) duration prolongation, resulting in an amplified dispersion of repolarization (DOR) in the tissue, leading to an increased QT interval and recurrent VT in a structurally normal heart^5^,^6^. Although gene mutations have been suggested to increase the risk of VT in LQTS patients, how the altered genotype generates clinically arrhythmic phenotypes in patients remains unclear.

Mutations in the *CACNA1C* gene have been linked to rare forms of LQTS which is associated with excessive cellular Ca^2+^ entry through Ca_V_1.2 L-type calcium channel and VT^7^,^8^,^9^,^10^,^11^,^12^,^13^,^14^,^15^,^16^,^17^,^18^,^19^,^20^,^21^. In *CACNA1C* mutations, there is a 5731 G>C transversion, corresponding to a 1911 glycine to arginine substitution (p.G1911R) in Ca_V_1.2^16^. Although functional analysis in both wild-type and G1911R mutation conditions revealed an increased L-type calcium current (*I*_*CaL*_) and suggested the mutant ionic promoted VT, this needs to be addressed directly^16^. Based on previous experimental data, computational models provided further analysis and physiological insights of genotype-phenotype associations^22^. In detail, modeling studies in *CACNA1C* mutations have shed light on the cellular and tissue-level mechanisms of arrhythmias^10^,^17^,^19^,^23^,^24^,^25^,^26^. It has been demonstrated that *CACNA1C* mutations caused a gain-of-function of Ca_V_1.2 L-type calcium channel, leading to an increased calcium influx, resulting in a dramatic AP prolongation^19^,^23^,^26^ and afterdepolarization-triggered activity^10^,^17^,^24^,^25^. They also steepened the action potential duration restitution (APDR) curve, disrupted rate-dependent cardiac excitation dynamics and promoted the development of alternans^24^. *CACNA1C* mutations amplified DOR and increased membrane potential spatial gradients across the tissue^24^,^25^. *CACNA1C* mutations prolonged QT interval^24^,^25^ and produced T-wave alternans and T-wave inversion^24^ in electrocardiography (ECG). Although simulation studies interpreted the effects of *CACNA1C* mutations on cellular functions, however, the effects of of *CACNA1C* mutations on cardiac excitation dynamics, characteristics of ECG and the dynamics of spiral waves (VT in human ventricle) have not been investigated in these studies^24^. In addition, as there is no accurate animal model of the G1911R *CACNA1C* mutation to date, the underlying mechanisms of VT genesis and maintenance are not fully understood. Consequently, the aim of this study was to quantify the pro-arrhythmogenic effects of the G1911R mutation in the human ventricular at cell, tissue strand, tissue sheet and whole organ levels by utilizing a computational modeling approach.

In this study, we hypothesized that the increased *I*_*CaL*_in the G1911R mutation augmented repolarization dispersion through the transmural ventricular wall with intrinsic repolarization heterogeneity, leading to an increased tissue’s vulnerability to generate unidirectional conduction block of excitation waves, facilitating the genesis of VT. In this process, the midmyocardial (MCELL) region severed as an excitable obstacle for stabilizing and sustaining VT. To test these hypothesizes, a human ventricular model including specific changes of *I*_*CaL*_ kinetics induced by the G1911R *CACNA1C* mutation was developed to investigate changes of *I*_*CaL*_ on AP, APD and effective refractory period (ERP). A one-dimensional (1D) strand model was then constructed to quantify the pro-arrhythmogenic effects of *CACNA1C* G1911R mutation on APD heterogeneity, membrane potential heterogeneity, electrocardiograph (ECG), vulnerable window (VW) and DOR. Finally, idealized two-dimensional (2D) and realistic three-dimensional (3D) models with a MCELL island were built to simulate the initiation and maintenance of re-entry and examine effects of G1911R mutation on the genesis of VT.

## Results

### Effects of *CACNA1C* G1911R mutation on single cell APs

**Figure 1:**
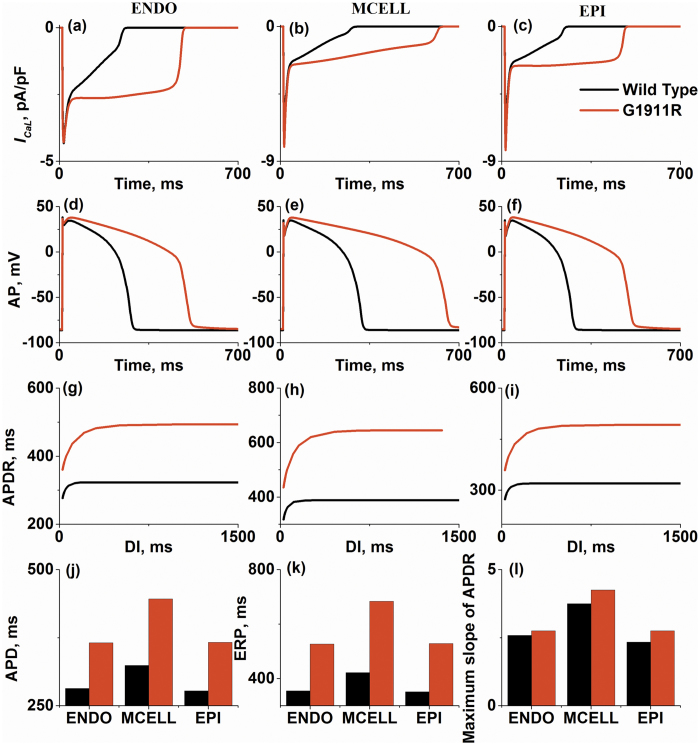
Simulations of *I*_*CaL*_current traces, action potentials (AP), action potential duration (APD), effective refractory period (ERP) and action potential duration restitution (APDR) under wild-type and G1911R conditions for ENDO cell, MCELL cell and EPI cell. (**a**–**c**) *I*_*CaL*_amplitude during AP under wild type and G1911R conditions for ENDO cell (**a**), MCELL cell (**b**) and EPI cell (**c**). (**d**–**f**) Simulated AP profiles under wild type and G1911R conditions for ENDO cell (**d**), MCELL cell (**e**) and EPI cell (**f**). (**g**–**i**) APDR curves under wild type and G1911R conditions for ENDO cell (**g**), MCELL cell (**h**) and EPI cell (**i**). (**j**) Transmural APD difference between wild type and G1911R conditions. (**k**) Transmural ERP difference between wild type and G1911R conditions. (**l**) Maximum slope difference of APDR curves between wild type and G1911R conditions.

Figure 1 shows that increased *I*_*CaL*_due to the *CACNA1C* G1911R mutation prolonged human ventricular APs. For endocardial cells (ENDO), the G1911R mutation significantly increased *I*_*CaL*_(Fig. 1a), which prolonged plateau of AP (Fig. 1d), APD (Fig. 1j) and ERP (Fig. 1k).

Similar results were observed in MCELL (Fig. 1b,e,h) and epicardial cells (EPI) (Fig. 1c,f,i). It is worthwhile to point out that the G1911R mutation prolonged APD in both MCELL and EPI (Fig. 1j,k), but to different extents. The APD prolongation in MCELL is more significant than that in other cell types. Under wild-type condition, the APD differences between ENDO and MCELL (ENDO-MCELL) and between EPI and MCELL (EPI-MCELL) are about ~50 ms. However, the APD difference in ENDO-MCELL and EPI-MCELL cells in the G1911R mutation went up to ~100 ms. Similar to APD prolongation, the G1911R mutation also increased ERP as shown in Fig. 1k. The increases of APD and ERP were inhomogeneous for the three different cell types, indicating an augmented transmural heterogeneity of APD across the ventricle wall in the G1911R mutation condition, leading to an increased dispersion of repolarisation.

To further investigate the effect of the G1911R mutation on cardiac restitution properties, APDR was calculated under wild-type condition and compared with that under the G1911R condition. Figure 1g–i show that the G1911R mutation produced an upward shift of APDR curves due to APD prolongation and steepened the APDR curve in ENDO, MCELL and EPI cells. The maximal slope of APDR curves under G1911R condition were also increased (Fig. 1l). The simulation results suggested an increase in ventricular APD rate adaptation, which may increase the instability of excitation waves^27^,^28^, leading to formation of re-entrant excitation waves^29^,^30^, which is consistent with recurrent VT in patients^16^.

In summary, the results in single cell simulations demonstrated that the G1911R mutation prolonged APD, increased electrical heterogeneity among different cell types and augmented the maximal slope of APDR, all of which plays an important role in predisposing to VT.

### Effects of *CACNA1C* G1911R mutation on ECG

**Figure 2:**
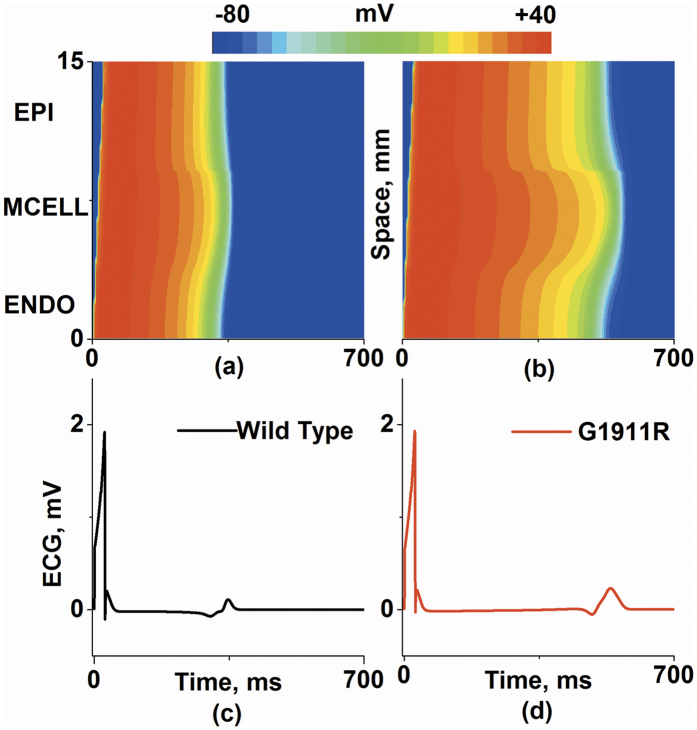
Space-time plots of propagating excitation wave and corresponding pseudo-ECG. (**a**,**b**) Color mapping of membrane potential along the 1D transmural ventricular strand under wild type (**a**) and G1911R (**b**) conditions. (**c**,**d**) Pseudo-ECGs corresponding to space-time plot of propagating excitation wave under wild type (**c**) and G1911R (**d**) conditions.

Propagating excitation waves and ECG traces were calculated to investigate effects of G1911R mutation at tissue strand level. As shown in Fig. 2, a propagating excitation wave was initiated by applying a supra-threshold stimulus to the ENDO end (Fig. 2a,b). The pseudo-ECGs were computed under wild-type (Fig. 2c) and G1911R (Fig. 2d) conditions. The G1911R mutation prolonged QT interval, elevated T wave and enlarged T wave width. In detail, the QT interval and T wave width were prolonged progressively from 367.8 ms and 20.3 ms under the wild type condition to 577 ms and 41.63 ms under the G1911R condition, respectively. In addition, these results are consistent with the major features of ECG signals recorded from patients^11^,^16^.

**Figure 3:**
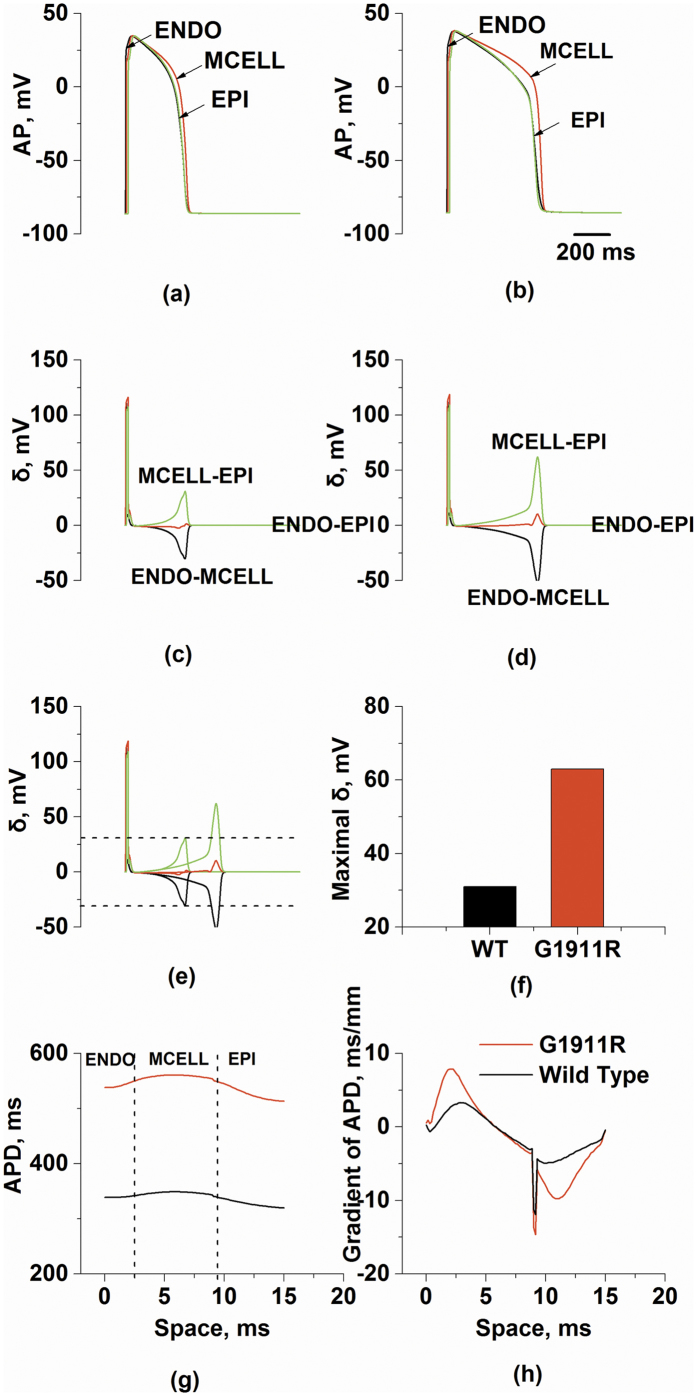
APs for ENDO cell, MCELL cell and EPI cell, membrane potential heterogeneity (δ) between transmural cells, transmural repolarization dispersion and its spatial gradient in 1D transmural strand for wild type and G1911R conditions. (**a**) ENDO, MCELL and EPI APs under the wild type condition. (**b**) ENDO, MCELL and EPI APs under the G1911R condition. (**c**) Membrane potential heterogeneity (δ) under the wild type condition. (**d**) Membrane potential heterogeneity under the G1911R condition. (**e**) Membrane potential heterogeneity difference between wild type and G1911R conditions during APs. (**f**) Measured maximum δ between wild type and G1911R conditions during repolarization. (**g**) Spatial distribution of APD on 1D transmural strand under wild type and G1911R conditions. (**f**) Spatial gradient of APD on 1D transmural strand between wild type and G1911R conditions.

To illustrate mechanisms underlying ECG variations in the G1911R mutation condition, the membrane potential heterogeneity (δ) among cell types and transmural repolarization dispersion across the intact 1D strand were investigated. Simulated ENDO, MCELL and EPI APs under wild-type and G1911R conditions are shown in Fig. 3a,b and corresponding δ plots between different cell types are shown in Fig. 3c,d. The maximum δ between ENDO and MCELL regions and between MCELL and EPI regions during repolarization were both enlarged under the G1911R condition (Fig. 3e,f). As the spatial gradient of membrane potential contributed to the ECG, the increased maximum spatial gradient during repolarization might account for the augmented T wave amplitude seen in Fig. 2. In addition to membrane potential heterogeneity, the G1911R mutation also augmented repolarization time (Fig. 3g) and increased the maximum APD spatial gradient (Fig. 3h). As repolarization time is associated with QT interval and DOR reflects T wave width, altered *I*_*CaL*_due to the G1911R mutation might account for the observed changes of ECG in patients with the G1911R mutation.

### Effects of *CACNA1C* G1911R mutation on tissue vulnerability to unidirectional conduction block.

**Figure 4:**
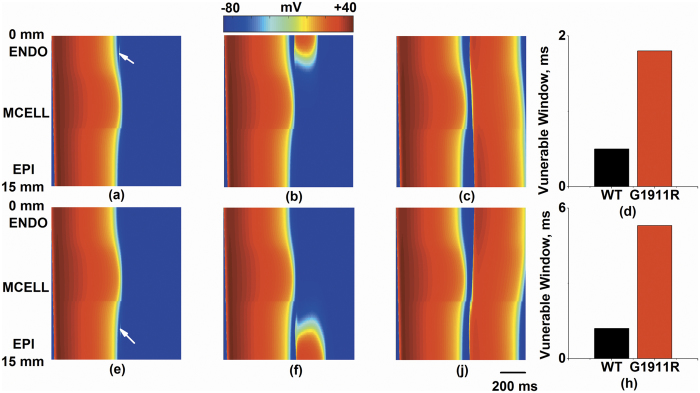
Measured width of vulnerable window and space-time plot of propagating excitation wave in response to a premature test stimulus at various time interval (Δ*t*) between S1 and S2 stimulus under G1911R condition. (**a**–**d**) A premature test stimulus applied at ENDO part (marked in the figure by arrow). (**a**) Bidirectional block with Δ*t* = 627 ms. (**b**) Unidirectional block with Δ*t* = 627.5 ms. (**c**) Bidirectional conduction with Δ*t* = 631 ms. (**d**) Measured width difference of vulnerable window at ENDO part between wild type and G1911R conditions. (**e**–**h**) A premature test stimulus applied at EPI part. (**e**) Bidirectional block with Δ*t* = 629 ms. (**f**) Unidirectional block with Δ*t* = 630 ms. (**g**) Bidirectional conduction with Δ*t* = 635 ms. (**h**) Measured width difference of vulnerable window at EPI part between wild type and G1911R conditions.

Since unidirectional conduction block increases susceptibility to VT, we quantified VW to examine effects of the G1911R mutation on tissue vulnerability to unidirectional conduction block. Figure 4 shows the excitation wave in response to a test stimulus which was applied in different portions of the fiber at different time delay (*Δt*) under the G1911R condition. In the ENDO region (marked in the Fig. 4a by arrow), a test stimulus could not re-excite the tissue at an early time delay (*Δt* = 627 ms) when the tissue did not recover from excitation, generating a bidirectional conduction block (Fig. 4a). When a time delay was within the VW (*Δt* = 627.5 ms), the tissue surrounding the test stimulus site partially recovered. In this condition, an excitation wave propagated towards recovered tissue and was blocked by unrecovered tissue, generating a unidirectional conduction block (Fig. 4b). When a time delay was sufficiently late and out of the VW (*Δt* = 631 ms), all the tissue had fully recovered, therefore the surrounding tissue were excitable, generating a bidirectional conduction (Fig. 4c). In detail, the measured VW in the ENDO region increased from 0.5 ms under the wild-type condition to 1.8 ms under the G1911R condition (Fig. 4d).

Similar results were shown in the EPI region. Bidirectional conduction block, unidirectional conduction block and bidirectional conduction of re-excitation waves in the EPI region (marked in the Fig. 4e by arrow) were evoked at *Δt* = 629 ms, 630 ms and 635 ms, respectively, as shown in Fig. 4e–g. In the EPI region, the VW increased by 200% (from 1.8 ms to 5.4 ms) in the G1911R condition (Fig. 4h). In addition, there was no unidirectional block observed in the MCELL region under wild-type and G1911R conditions.

Thus, the simulation results indicated that the G1911R mutation augmented tissue vulnerability to unidirectional conduction block by increasing transmural repolarization dispersion.

### Effects of *CACNA1C* G1911R mutation on spiral wave in idealized 2D model

**Figure 5:**
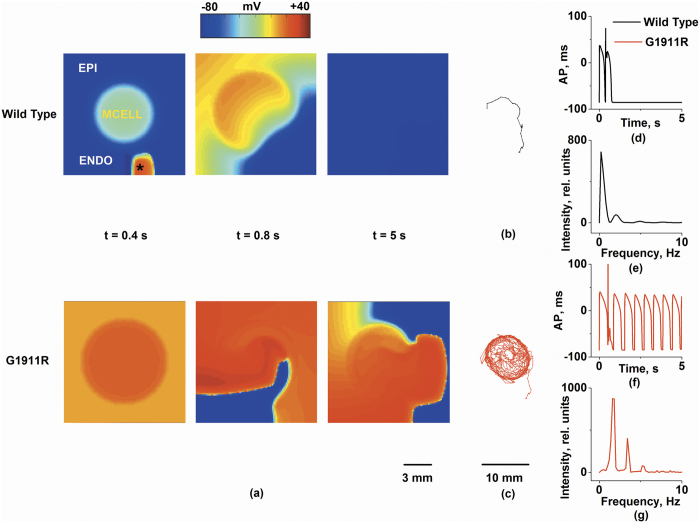
Simulation of spiral waves in a 2D ventricular tissue which included a subendocardium (ENDO), a subepicardium (EPI), and a midmyocardium (MCELL) as indicated. Frames from the 2D simulation under the wild type and G1911R conditions at *t* = 0.4 s, *t* = 0.8 s and *t* = 5 s are shown in Fig. 5a. Trajectories of the re-entrant wave tip for wild type and G1911R conditions are shown in Fig. 5b,c, respectively. Recorded time series of the AP (**d**,**f**) of a cell (marked in the figure by asterisk) dominant frequencies (**e**,**g**) within 5s are were compared.

Spiral waves were initiated by applying a test stimulus within the VW at the ENDO region (Fig. 5). In this case, since the MCELL region has slower repolarization than the ENDO and EPI regions, the longer ERP of the MCELL region gave rise to a unidirectional conduction towards the ENDO side, forming an excitable obstacle to conduction. Then, the excitation wave was attracted to the excitable obstacle and generated a spiral wave. Figure 5a shows spiral waves propagation under wild-type and G1911R conditions. Under the wild-type condition, the initiated spiral wave terminated spontaneously (Fig. 5b). However, the re-entrant wave in the G1911R group was stable and persistent at a high rate and the tip of the spiral wave was attracted into the MCELL region (Fig. 5c). The measured life span of a spiral wave and the peak frequency of local electrical activity were 0.9 s and 0.78 Hz in the wild type group (Fig. 5d,e) respectively, and 5 s and 2.5 Hz in the G1911R group (Fig. 5f,g) respectively. Detailed movies of spiral wave propagation in idealized 2D model under wild-type and G1911R conditions are shown in Supplementary Videos S1 and S2 respectively.

Thus, the simulation results in idealized 2D model indicated that the G1911R mutation facilitated the genesis of reentry by augmenting tissue vulnerability to unidirectional conduction block.

### Arrhythmogenic substrates in the *CACNA1C* G1911R mutation in realistic 3D left ventricle

**Figure 6:**
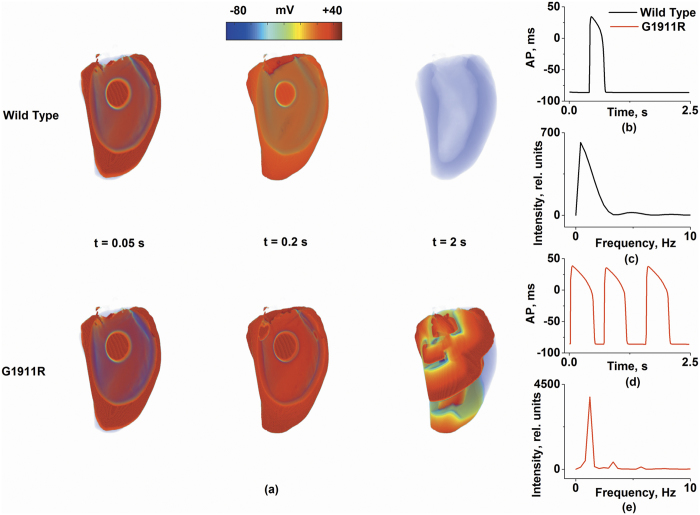
Simulation of scroll waves in a 3D virtual left ventricular with a MCELL island. Frames from the 3D simulation under wild type and G1911R conditions at *t* = 0.05 s, *t* = 0.2 s and *t* = 2 s are shown in Fig. 6a. Localized potentials (**b**,**d**) and dominant frequencies (**c**,**e**) within 5 s are were compared.

In the realistic 3D left ventricular model, the dynamics of spiral waves were studied to reveal the effects of the G1911R mutation on VT. In detail, excitation waves in all groups were initiated by employing a test stimulus at the ENDO region in the 3D human left ventricular model with a MCELL island (Fig. 6a). Under the wild-type condition, the excitation wave was not attracted to the MCELL region and meandered in a large area of the tissue, leading to self-termination at 0.7 s and manifesting as a single cycle of AP in Fig. 6b. Under the G1911R condition, the excitation wave was attracted to the MCELL island and generated self-maintained spiral waves (Fig. 6d). The life span of excitation wave and the peak frequency of local electrical activity were 0.7 s and 1.65 Hz in the wild-type group (Fig. 6b,c). However, the re-entrant wave persisted throughout the whole simulation process (2.5 s) under the G1911R condition (Fig. 6d). Meanwhile, the dominant frequency in the G1911R case was much higher (~2.4 Hz) than that under the wild-type condition (Fig. 6e). Detailed movies of spiral wave propagation in realistic 3D model under wild-type and G1911R conditions are shown in Supplementary Videos S3 and S4 respectively.

The simulation results in realistic 3D model indicated that the G1911R mutation facilitated and perpetuated organ-scale VT by increasing repolarization dispersion.

## Discussion

Mutations in *CACNA1C* are associated with life-threatening arrhythmias and some patients with this type of mutations may have an episode of VT^7^,^8^,^11^,^15^,^16^,^19^. In the G1911R mutation, a patient with the heterozygotic G1911R mutation demonstrated a QT interval of 520 ms at age 5 years and suffered from VT^16^. Although it has been reported that the G1911R mutation caused a gain-of-function of Ca_V_1.2, mechanisms by which the *CACNA1C* G1911R mutation facilitate and perpetuate organ-scale VT remain unclear. In this study, we quantified pro-arrhythmogenic effects of the G1911R mutation in the human ventricular at cell, tissue strand, tissue sheet and whole organ levels by utilizing a computational modeling approach. We demonstrated that the QT interval was prolonged from 367.8 ms under the wild-type condition to 577 ms under the G1911R condition. Importantly, spiral waves were shown under the G1911R condition which is consistent with the clinically observable VT. Our results indicated that the G1911R mutation (i) increased *I*_*CaL*_ by changing voltage-dependence of activation, voltage-dependence of inactivation and the time constant of inactivation; (ii) prolonged APD and ERP and steepened APD restitution curves, leading to enhanced rate-dependent adaptation of APD; (iii) prolonged QT interval, increased T wave amplitude and lengthened *T*_*peak*_–*T*_*end*_ duration; (iv) augmented membrane potential and APD heterogeneity, increased repolarization dispersion and VW for unidirectional block and promoted the formation of spiral waves; (v) the MCELL island formed an excitable obstacle which anchored the spiral wave and stabilized re-entry, leading to more frequent ventricular excitations. Clinically, electrical heterogeneity was also reported to be enhanced in cardiac tissue in patients with LQTS^5^,^6^. Taken together, our data demonstrates that increased repolarization dispersion may be the causative link between the altered genotype and the clinical arrhythmic phenotypes in patients.

As VT remains one of the most common causes of sudden cardiac death, many studies have been undertaken to analyze its initiation. Particularly, observations in patients with the G1911R mutation provided insight into the mechanisms of VT^16^. Macroscopic reentry and VT have been found to occur in the patients’ heart with the G1911R mutation^16^. In addition, LQTS is characterized by regional APD prolongation^5^,^6^. In the present paper, we focused on the initiation and maintenance of arrhythmias in transmural ventricular tissue, which are usually associated with reentry. Our study suggests that there are two facts mainly contributed to the pro-arrhythmic effects of G1911R mutation.

The first one is the increased repolarization time and ERP in G1911R mutation. In the G1911R mutation, a defect in voltage-dependent inactivation (VDI) increased Ca_V_1.2 window current and prolonged the plateau phase of the action potential, producing excessive APD prolongation. Consequently, this augmented repolarization time (QT interval) of tissue and increased ERP, facilitating conduction block of excitation waves.

The other fact contributed to the pro-arrhythmic effects of G1911R mutation is the increased electrical heterogeneity of the tissue, reflected by the increased repolarization dispersion. Our findings support the notion that enhanced repolarization dispersion is proarrhythmic^24^,^25^. On the one hand, the G1911R mutation augmented transmural heterogeneity of APD as the APD prolongation among cell types were different. The difference of repolarization among cell types augmented transmural DOR under the G1911R condition. The greater dispersion of repolarization significantly prolonged *T*_*peak*_–*T*_*end.*_ Therefore, the G1911R mutation caused greater repolarization gradients between different cell regions in cardiac tissues and consequently increased the VW facilitating the genesis of VT. On the other hand, membrane potential heterogeneity was also enhanced by the G1911R mutation. The membrane potential gradients (the temporal gradients) between ENDO and MCELL and between EPI and MCELL during repolarization were augmented respectively in the G1911R mutation condition. Thus, the greater dispersion of membrane potential augmented T wave amplitude. In summary, the increased dispersion of repolarization due to the G1911R mutation led to augmented spatial and temporal gradients, together with a steeper APDR, increasing susceptibility to alternans development which was implicated in life-threatening arrhythmia by promoting wave break^31^.

For the idealized 2D model, the transmural ventricular tissue is composed by ENDO, MCELL and EPI. Many studies have been undertaken to analysis the role of MCELL in LQTS arrhythmogenesis^5^,^6^,^32^,^33^,^34^. Particularly, experimental studies in animals and patients with LQTS have shown how the size and distribution of MCELL in transmural ventricular tissue became arrhythmogenesis^5^,^6^,^32^. Regional APD prolongation was observed^6^ and size of the region was measured^5^ in patients with LQTS. Most importantly, islands of MCELL, which provided the distribution of MCELL in transmural tissue, were observed in canine wedge^32^. In this paper, we constructed an idealized 2D transmural ventricular tissue model based on the size and distribution of the MCELL region. On the one hand, the width of 2D model is consistent with the normal range of human transmural ventricular width^35^. In the present simulations, the size of MCELL island was similar to the area of prolonged APD region in human heart^5^,^34^. Our model gives a theoretical frame and supports our hypothesis on the proarrhythmic effects of increased DOR, particularly resulting from the MCELL island. Indeed, we did observe a prolonged APD region under the G1911R mutation condition and the region (the MCELL island) formed an excitable obstacle which can anchor spiral waves, forming a novel mechanism of generating VT by increased regional heterogeneity in the G1911R mutation condition.

Spatial heterogeneous APD prolongation created a relatively stable and persistent excitable obstacle in the MCELL region. The maximal difference in APD/ERP among three cell types was determining such an excitable obstacle. The G1911R mutation significantly increased the maximal difference in APD/ERP among three cell types, resulting in a sustained functional excitable obstacle. The spiral wave was attracted to the excitable obstacle and its tip meandered around the obstacle, thereby leading to stable and persistent re-entry under the G1911R condition, which is consistent with regional heterogeneity-induced arrhythmogensis^32^,^33^,^34^,^36^,^37^. The results support that unique distribution of MCELL underlies reentrant mechanism in LQTS^36^,^33^. It is particularly noted that unique circular distribution of MCELL was directly responsible for conduction block and self-sustained transmural re-entrant circuits underlying VT. The-entrant wave in our simulation was not identical to that in 2D models with a strip of MCELL used in previous studies^28^,^33^,^35^. Thus, excitation wave propagation for wild-type and G1911R conditions in idealized 2D with ENDO, MCELL and EPI layers was investigated and shown in Supplementary Videos S5 and S6 respectively. Similar to the circular distribution, a strip of MCELL initiated and sustained spiral waves under the G1911R condition. In addition, the results in realistic 3D model under the G1911R condition also showed sustained spiral waves with a higher dominant frequency and a longer lifespan (Detailed movies of spiral wave propagation in realistic 3D model with ENDO, MCELL and EPI layers under wild-type and G1911R conditions are shown in Supplementary Videos S7 and S8). Thus, the simulation results in idealized 2D and realistic 3D models confirmed that the G1911R mutation facilitates and perpetuates organ-scale VT by enhancing heterogeneity.

In addition, augmented *I*_*CaL*_ has previously been implicated in disrupting cardiac dynamics and promoting alternans by steepening action potential duration restitution^24^. Also, it was shown that increased dispersion of repolarization was required for unidirectional conduction block under the G406R/S402R mutation condition^25^. Our findings demonstrated that in the G911R mutation, the altered *I*_*CaL*_ not only steepened action potential duration restitution but also increased DOR, which promoted initiation and maintenance of cardiac arrhythmias. Therefore, our study implicates augmented *I*_*CaL*_ due to *CACNA1C* mutations in tachycardia arrhythmogenesis.

Limitations of ten Tusscher *et al.* have been addressed elsewhere^28^,^35^,^38^,^39^. Although our model successfully reproduced spiral waves (VT in human ventricle) under the G1911R condition, it has several limitations which need to be addressed in future study. First, the clinically observable arrhythmic phenotypes in patients often occur in the case of increased sympathetic tone^31^. Due to the lack of precise computational models of multiple signaling pathways, the relative contribution of active specific targets, such as calmodulin-dependent protein kinase II, β-adrenergic receptor, protein kinase A, etc.^10^,^25^,^31^ to the pro-arrhythmic effects of the G1911R mutation were not included. Second, it should be noted that the generation of afterdepolarization has been proposed as one of the arrhythmic mechanisms in *CACNA1C* mutations^10^,^17^,^24^,^25^. However, single cell simulations did not exhibit the occurrence of afterdepoloarization in our models and afterdepoloarization may be associated with multiple signaling pathways^10^,^25^. Third, the *CACNA1C* mutation increased intracellular calcium concentration, which influenced cardiac mechanics. In this study, cardiac mechanics was not included in our model and its effect on reentry was not studied^40^,^41^. Fourth, it should be noted that the distribution and population of each cell type in cardiac tissues could influence the cardiac dynamics and there is no detailed experimental data to modify the model. In this study, the related parameters were chosen according to previous studies^28^,^35^,^42^. Although it is important to make clear the limitations of the models in this study, they do not alter our conclusion on that the *CACNA1C* G1911R mutation promotes initiation and maintenance of cardiac arrhythmias.

In conclusion, the G1911R mutation facilitates and perpetuates organ-scale VT by increasing repolarization dispersion. The findings of this study provide new insights into the mechanism underlying the development and maintenance of VT in patients with *CACNA1C* G1911R mutation and emphasize the critical role of MCELL in the genesis of ventricular arrhythmias.

## Methods

### Mathematical model

Using the experimental data of the changes of *I*_*CaL*_in the *CACNA1C* G1911R mutation^16^, the TNNP models were modified for simulating human ventricular cellular functions (for details see Supplementary Table S1)^38^. The simulated and experimental data of *I*_*CaL*_ under wild-type and G1911R conditions are well matched as shown in Supplementary Table S2. The TNNP model was chosen in this study as it includes *I*_*CaL*_ from human ventricular myocytes and reproduces APs of human endo-cardial (ENDO), middle-cellular (MCELL) and epi-cardial (EPI). These models were shown to be well-suited to study VT^29^,^30^,^35^,^39^.

The ventricular cell models were then integrated to construct tissue models with special tissue geometry and to simulate the wave propagation. The tissue models can be described as


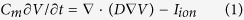


where *D* is the electronic diffusion modeling gap junction coupling, *V* (mV) is the membrane potential, *C*_*m*_(pF) is the membrane capacitance, *I*_*ion*_ (pA/pF) is the sum of ionic currents, 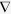
is the 1D, 2D or 3D gradient operator and *t* (ms) is time. For definitions of each component of *I*_*ion*_, please see ten Tusscher *et al.*^38^. The diffusion coefficient (*D*) across the fibers (transmural strands) is four times less than that along the fibers^34^, which is set to be 0.154 mm^2^/ms, producing a planar wave with the conduction velocity of 0.715 mm/ms along the fiber direction^38^. In addition, *D* at the EPI-MCELL border is 5-fold smaller^43^.

Multi-scale ventricular models were developed to study excitation wave propagation. A 15 mm 1D transmural strand was constructed consisting of 25% ENDO cells, 35% MCELL cells and 40% EPI cells. The proportion of cells is similar to those used in other studies^28^,^35^. Because the body surface potential mapping in LQTS patients has demonstrated a marked increase regional heterogeneity in repolarization^6^, we constructed the 2D idealized transmural model of the human ventricular tissue, using the same proportion of MCELL cell as that in the 1D strand and similar size of the MCELL region according to experimental and simulated studies^5^,^6^. The distribution of the MCELL island of idealized 2D and realistic 3D models is depicted in Supplementary Figure S1. The size of the MCELL island is used in the models based on the heterogeneous region following experimental and simulation studies^5^,^6^. The anterior myocardial wall in the left ventricle encompassed a MCELL island. Transmurally, tissue included a MCELL island and the subepi- and subendocardium. The idealized 2D transmural ventricular tissue sheet (15 mm × 15 mm) with a MCELL island (π × 5.1 mm × 5.1 mm, a size similar to the heterogeneity region measured in the human heart and that used in a previous simulation study^5^,^6^) was simulated and the realistic 3D left ventricular model, including a MCELL island (4/3 × π × 5.1 mm × 5.1 mm × 5.1 mm) was constructed.

### Single cell simulations: AP, APD and ERP

Single cell APs were elicited with supra-threshold stimuli with the amplitude of −40 μA/cm^2^ and the duration of 0.5 ms. 30 stimuli were applied at a pacing cycle length (PCL) of 1000 ms to obtain stable AP profiles and the 31st AP was used in analysis. APD was measured at 90% repolarization. APDR was assessed by applying the standard stimulus (S1–S2) protocol. The basic cycle length is 1000 ms and the premature coupling interval (S1–S2) was shortened in steps before S2 was blocked^24^. The restitution curve (APDR) was obtained by plotting S2–induced APD against the last S1 diastolic interval (DI). Effective refractory period (ERP) was measured as the smallest premature coupling interval (S1–S2) for which the peak potential of the S2-induced AP reached 80% of the last S1-induced AP^28^,^35^.

### 1D tissue strand simulations: ECG, vulnerable window and dispersion of repolarization

The transmural excitation waves in the transmural ventricular fiber were initiated by supra-threshold stimuli (similar to that of AP simulations) applied to the ENDO end. Dispersion of repolarization (DOR) was measured as the time interval between the earliest and the latest repolarization times in the strand. The vulnerable window, in which excitation can propagate in one direction, is the time window between the time of bidirectional conduction block and that of bidirectional conduction. The method of Gima and Rudy^43^ was used to compute pseudo-ECG.

### Initiation of reentry in idealized 2D and realistic 3D models of human ventricle

Re-entry was initiated by applying a test stimulus within the VW at the ENDO region to evoke unidirectional propagation^28^,^35^. After initiation, re-entrant electrical activities in idealized 2D sheet was recorded for 5 s. The stability of the re-entry was assessed by the path and area of its tip. The method of Fenton and Karma^44^ was used to trace the tip meander path. Life span (LS) of the re-entry was computed as the time duration from initiation to dissipation of a spiral wave. Dominant frequencies of virtual monophasic AP profiles from a special site were computed using standard fast Fourier transformed techniques. The same methods were used in realistic 3D left ventricular model.

### Numerical methods

The partial differential equations were solved by an explicit forward Euler approximation. The time step (*Δt*) is 0.02 ms, and Δx, *Δy* and *Δz* are the space steps. The space steps are 0.15 mm for 1D simulations^28^,^35^, 0.2 mm for 2D simulations and 0.5 mm for 3D simulations^34^. Simulations were carried out on an Intel core i703930K 64-bit CPU system with 64 G memory. Efficient parallelization was implemented using GPU acceleration.

## Additional Information

**How to cite this article**: Bai, J. *et al.* Pro-arrhythmogenic effects of *CACNA1C* G1911R mutation in human ventricular tachycardia: insights from cardiac multi-scale models. *Sci. Rep.* **6**, 31262; doi: 10.1038/srep31262 (2016).

## Supplementary Material

Supplementary Information

Supplementary Video S1

Supplementary Video S2

Supplementary Video S3

Supplementary Video S4

Supplementary Video S5

Supplementary Video S6

Supplementary Video S7

Supplementary Video S8
