# Supplementary material for: Pro-arrhythmogenic effects of CACNA1C G1911R mutation in human ventricular tachycardia: insights from cardiac multi-scale models
Source: Sci Rep. 2016 Aug 9;6:31262. doi: 10.1038/srep31262 (PMC4977499; doi:10.1038/srep31262)
Supplement: Supplementary Information [file srep31262-s1.doc]

**Pro-arrhythmogenic effects of *CACNA1C* G1911R mutation in human ventricular tachycardia: insights from cardiac multi-scale models**

Jieyun Bai^1^, Kuanquan Wang^1*^, Qince Li^1^, Yongfeng Yuan^1^ & Henggui Zhang^1,2^

^1^ School of Computer Science and Technology, Harbin Institute Technology, Harbin, 150001, China

^2^ Biological Physics Group, School of Physics and Astronomy, University of Manchester, Manchester, M13 9PL, UK

^*^Correspondence and requests for materials should be addressed to W.K. ([wangkq@hit.edu.cn](mailto:wangkq@hit.edu.cn))

### Abstract

Here, we present Supplementary information for our manuscript.

### Appendix

***Development of the I_CaL_ model***: We modified *I_CaL_* [kinetics](http://www.baidu.com/link?url=TPCn8huib2Au1KY5EyZZZfjDBVMx9KOBvERsTysJ25MoQz9WMAEafcYThJhzNmn-Wb4gAin7jGadmwf4Wlt1R8Jvy2a_iOfw1kKcPrae9gO) to reproduce the effects of the G1911R mutation on Ca_V_1.2 (for details see Supplementary Table S1). Following ten Tusscher *et al* ^1^, *I_CaL_* was described as

$I_{C\text{a}L}=G_{CaL}dff_{Ca}4\frac{VF^{2}}{RT}\frac{Ca_{i}e^{2VF/RT}-0.341Ca_{O}}{e^{2VF/RT}-1}$ (1)

$d_{\infty}=\frac{1}{1+e^{(V_{a0.5}-V)/S_{a}}}$ (2)

$f_{\infty}=\frac{1}{1+e^{(V-V_{ina0.5})/S_{ina}}}$ (3)

$\tau_{f}=1125e^{-(V+27)^{2}/240}+\frac{165}{1+e^{(25-V)/10}}+T_{ina}$ (4)

where *d* is a voltage-dependent activation gate, *f* is a voltage-dependent inactivation (VDI) gate, *f_Ca_* is a calcium-dependent inactivation gate, *τ_f_* is the time constant of *f* gate, *Ca_i_* is the intercellular concentration of *Ca*^2+^, *Ca_o_* is the extracellular concentration of *Ca*^2^*^+^* and *T_ina_* is a constant parameter of *τ_f_*. *R*, *T* and *F* represent the gas constant, the temperature (310*K*) and Faraday constant.

Based on experimental data of Hennessey *et al*., *CACNA1C* G1911R mutation-induced changes in the equations for *I_CaL_* include: (i) the channel maximal conductance (*G_CaL_*) (ii) the half-activation voltage (*V_a0.5_*) and its slope factor (*S_a_*); (iii) the half-inactivation voltage (*V_ina0.5_*) and its slope factor (*S_ina_*); (iv) the inactivation time-constant (*τ_f_*). Compared with the wild type condition, maximal *I_CaL_* conductance augmented by 34%, the voltage-dependent steady-state activation curve shifted ~-5 mV and the steady-state VDI curve shifted ~+6 mV, thereby significantly increasing the Cav1.2 window current. Additionally, the mean tau of inactivation increased about 80 ms for G1911R compared with wild type, showing a defect in VDI^2^. These simulated changes of *I_CaL_* under the wild type and G1911R conditions well matched experimental data of Hennessey *et al.* (for details see Supplementary Table S2).

**Supplementary Table S1**. *CACNA1C* G1911R mutation-induced changes in *I_CaL_* channel kinetics

|  | Wild Type | G1911R | Original TNNP model |
| --- | --- | --- | --- |
| *G_CaL_* _(mm_^3^_/(ms_ *_μ_*_F))_ | 0.000119875 | 0.00014175 | 0.000175 |
| *f_Ca_* | 1 | 1 | _ |
| *V_a0.5_* _（_*_mV_*_）_ | -4.9 | -7.9 | -5 |
| *S_a_* _（_*_mV_*_）_ | 4.9 | 4.5 | 7.5 |
| *V_ina0.5_* _（_*_mV_*_）_ | -39.2 | -33.4 | 20 |
| *S_ina_* _（_*_mV_*_）_ | 7.6 | 6.7 | 7 |
| *T_ina_*_（_*_ms_*_）_ | 40 | 120 | 80 |

*Note:* Experimentally, Ba^2+^ has been widely used in lieu of Ca^2+^ to uncouple Ca^2+^ entry through the channel while allowing for investigation of voltage dependent properties without Ca^2+^ dependent inactivation. Using Ba^2+^ allows for a measure of purely VDI. In the simulated VDI protocols, the CDI is not permitted (achieved by clamping the CDI gate, *f_Ca_*, to 1).

**Supplementary Table S2**. Experimental and simulated changes of *I_CaL_* channel kinetics for wild type and G1911R conditions.

|  | Wild Type  (Experiment) | Wild Type (Simulated) | G1911R (Experiment) | G1911R (Simulated) |
| --- | --- | --- | --- | --- |
| Current Density (pA/pF) | -11.3 | -11.3 | -16.5 | -16.5 |
| *V*_0.5_ activation (mV) | -4.9 | -4.9 | -7.9 | -7.9 |
| *k* activation | 4.9 | 4.9 | 4.5 | 4.5 |
| *V*_0.5_ inactivation (mV) | -39.2 | -39.2 | -33.4 | -33.4 |
| *k* inactivation | 7.6 | 7.6 | 6.7 | 6.7 |
| Tau inactivation at 0 mV (ms) | 98.9 | 106 | 211.3 | 201 |
| Tau inactivation at 10 mV (ms) | 76.8 | 73.8 | 160.1 | 168 |

***Transmural models with a MCELL island:***

The TNNP cellular model was incorporated into idealized 2D and realistic 3D ventricular wall was composed of [endocardial](https://images.search.yahoo.com/search/images;_ylt=AwrSbgEpEmFXoysA9L1XNyoA;_ylu=X3oDMTByNWU4cGh1BGNvbG8DZ3ExBHBvcwMxBHZ0aWQDBHNlYwNzYw--?p=Endocardial&fr=sfp) and [endocardial](https://images.search.yahoo.com/search/images;_ylt=AwrSbgEpEmFXoysA9L1XNyoA;_ylu=X3oDMTByNWU4cGh1BGNvbG8DZ3ExBHBvcwMxBHZ0aWQDBHNlYwNzYw--?p=Endocardial&fr=sfp) layers and a midmyocardial island (shown in Supplementary Figure S1). Spiral waves were initiated by using an S1-S2 protocol. The entire tissue was stimulated once at the beginning of the simulation with S1^3, 4^. Then, an S2 stimulus was applied in the endocardium of the ventricle partly during the repolarization phase of a conditioning wave. The S2-evoked excitation wave propagates unidirectionally (shown in Supplementary Videos S1, S2, S3 and S4), leading to formation of reentrant excitation wave within the transmural wall.

**
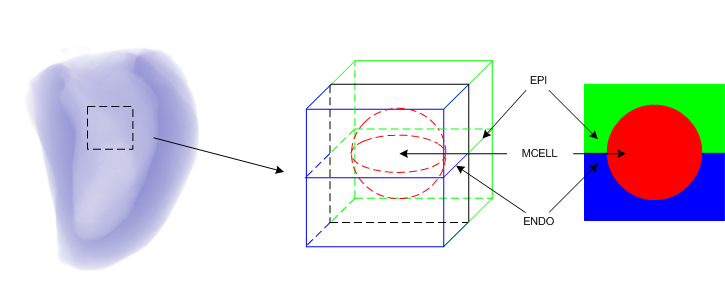
**

**Supplementary Figure S1**. Anterior and cross-sectional views of the human ventricular model showing a subendocardium (blue), a subepicardium (green), and a midmyocardium (red) with dimensions as indicated.

***Transmural models with ENDO, MCELL and EPI layers:*** The TNNP cellular model was incorporated into idealized 2D and realistic 3D ventricular wall was composed of three regions containing: 25% ENDO cells, 35% MCELL cells and 40% EPI cells. These proportions are similar to those used in other studies^5, 6^. 2D and 3D spiral waves were initiated by using an S1-S2 protocol. The S1 stimulus was applied to an ENDO zone that produced the activation timing sequence across the ventricular wall. Then, an S2 stimulus was applied in the epicardium of the left ventricle partly during the repolarization phase of a conditioning wave. The S2-evoked excitation wave propagates unidirectionally (shown in Supplementary Videos S5, S6, S7 and S8), leading to formation of reentrant excitation wave within the transmural wall.

### References

1. ten Tusscher, K.H.W.J., Noble, D., Noble, P.J. & Panfilov, A.V. A model for human ventricular tissue. *Am J Physiol-Heart C* **286**, H1573-H1589 (2004).
2. Hennessey, J.A.*, et al.* A CACNA1C Variant Associated with Reduced Voltage-Dependent Inactivation, Increased Ca(V)1.2 Channel Window Current, and Arrhythmogenesis. *Plos One* **9**(2014).
3. de Lange, E., Xie, Y. & Qu, Z. Synchronization of early afterdepolarizations and arrhythmogenesis in heterogeneous cardiac tissue models. *Biophysical journal* **103**, 365-373 (2012).
4. Sato, D.*, et al.* Synchronization of chaotic early afterdepolarizations in the genesis of cardiac arrhythmias. *Proc Natl Acad Sci U S A* **106**, 2983-2988 (2009).
5. Zhang, H., Kharche, S., Holden, A.V. & Hancox, J.C. Repolarisation and vulnerability to re-entry in the human heart with short QT syndrome arising from KCNQ1 mutation - A simulation study. *Prog Biophys Mol Biol* **96**, 112-131 (2008).
6. Adeniran, I., El Harchi, A., Hancox, J.C. & Zhang, H.G. Proarrhythmia in KCNJ2-linked short QT syndrome: insights from modelling. *Cardiovasc Res* **94**, 66-76 (2012).

### Supplementary Video Captions

**Supplementary Video S1: WT reentry in 2D transmural ventricular sheet with a MCELL island.** Re-entrant spiral waves generated by the application of a test stimulus into the partially recovered ENDO region of an excitation wave after a delay of 364 ms from the initial wave stimulus under the wild-type condition. The induced spiral waves could not be attracted by the MCELL island, rotated around the ventricle boundary and self-terminated within 1000 ms.

**Supplementary Video S2: G1911R reentry in 2D transmural ventricular sheet with a MCELL island.** Re-entrant spiral waves generated by the application of a test stimulus into the partially recovered ENDO region of an excitation wave after a delay of 628 ms from the initial wave stimulus under the wild-type condition. The induced spiral wave persisted and rotated around the MCELL island throughout the duration of the simulation period of 5000 ms.

**Supplementary Video S3: WT reentry in 3D left ventricular model with a MCELL island.** A spiral wave was initiated by the application of a stimulus into a local recovered ENDO region, conducted to the whole left ventricle and did not generated the spiral waves under the Wild type condition.

**Supplementary Video S4: G1911R reentry in 3D left ventricular model with a MCELL island.** A spiral wave was initiated by the application of a stimulus into a local recovered ENDO region, rotated around the MCELL island and generated the self-sustained spiral waves under the G1911R condition.

**Supplementary Video S5: WT reentry in 2D transmural ventricular sheet with ENDO, MCELL and EPI layers.** Re-entrant spiral waves generated by the application of a test stimulus into the partially recovered EPI region of an excitation wave after a delay of 348 ms from the initial wave stimulus under the Wild type condition. The spiral wave self-terminates within 1000 ms.

**Supplementary Video S6: G1911R reentry in 2D transmural ventricular sheet with ENDO, MCELL and EPI layers.** Re-entrant spiral waves generated by the application of a test stimulus into the partially recovered EPI region of an excitation wave after a delay of 629 ms from the initial wave stimulus. The spiral wave persists under the G1911R condition.

**Supplementary Video S7: WT reentry in 3D left ventricular model with ENDO, MCELL and EPI layers.** A spiral wave was initiated by the application of a test stimulus into the partially recovered EPI region after a delay of 350 ms from the initial wave stimulus. The spiral wave self-terminates within 2000 ms.

**Supplementary Video S8: G1911R reentry in 3D left ventricular model with ENDO, MCELL and EPI layers.** A spiral wave was initiated by the application of a test stimulus into the partially recovered EPI region after a delay of 525 ms from the initial wave stimulus. The spiral wave persists under the G1911R condition.
